# Supplementary material for: Association between Early Mobilization in the ICU and Psychiatric Symptoms after Surviving a Critical Illness: A Multi-Center Prospective Cohort Study
Source: J Clin Med. 2022 May 5;11(9):2587. doi: 10.3390/jcm11092587 (PMC9099642; doi:10.3390/jcm11092587)
Supplement: Supplementary file 1 [file jcm-11-02587-s001.zip › jcm-1655119-supplementary.pdf]

Table S1. Exclusion criteria.

Exclusion Criteria	Details
Age < 18 years	
Unable to walk independently before ICU admission	Patients who require a wheelchair other than a cane or other walking assistance prior to admission or help from others to walk were considered unable to walk independently.
Neurological complications	Neurological complications include cerebral infarction, cerebral hemorrhage, acute subdual hematoma, acute epidural hematoma, traumatic subarachnoid hemorrhage, and encephalitis.
Lack of communication skill because of pre-existing mental diseases	Mental diseases include depression, anxiety, schizophrenia, dementia, cerebral infarction, cerebral hemorrhage, and alcoholism.
In terminal state or end of life	

Table S2. Early Mobilization Protocol [a].

Level 1 Respiratory	Level 2 HOB	Level 3 Sitting	Level 4 Standing	Level 5 Walking
RASS−5～−3	RASS ≥ −3	RASS ≥ −1	RASS ≥ 0	RASS ≥ 0
**Physical therapy**<br>Passive ROM exercise<br>Respiratory physical therapy	**Physical therapy**<br>Positioning<br>Passive ROM exercise<br>Active ROM exercise<br>Respiratory physical therapy<br>continuous lateral rotation therapy	**Physical therapy**<br>Positioning<br>Passive ROM exercise<br>Active ROM exercise<br>Sitting on the edge of bed<br>Rising from the supine position	**Physical therapy**<br>Positioning<br>Passive ROM exercise<br>Active ROM exercise<br>Standing at side of bed<br>Stand and pivot to a chair	**Physical therapy**<br>Positioning<br>Passive ROM exercise<br>Active ROM exercise<br>Walk with assistance<br>Walk independently
**Positioning**<br>Posture change<br>HOB ≤ 45 degrees	**Positioning**<br>Posture change<br>HOB ≥ 60	**Positioning**<br>Posture change<br>HOB ≥ 60	**Positioning**<br>Posture change<br>HOB ≥ 60	**Positioning**<br>Posture change<br>HOB ≥ 60
**Step up criterion**<br>Oxygenation/ hemodynamic stability<br>Can withstand posture change<br>Can withstand HOB ≤45 degrees	**Step up criterion**<br>Can withstand supplementary motion of physical therapy<br>Can withstand HOB ≤60 degrees<br>Anti-gravity movement possible	**Step up criterion**<br>Can endure the active movement of physical therapy<br>Can withstand HOB ≤60 degrees<br>Can withstand sitting on the edge bed	**Step up criterion**<br>All exercise can be carried out<br>Can withstand partial weight standing	**Step up criterion**<br>Increase walking distance gradually
**Step up criterion to level 3 or higher are defined as**<br>RASS: −2 to +1, BPS ≤ 3 or NRS ≤ 5, SpO_2 ≥ 90%, FIO_2 < 0.6, PEEP < 10cmH_2O, respiratory rate: <35 times/min, mean blood pressure ≥ 65 mmHg, heart rate: 50 to 120 times/min, there were no new arrhythmias, no additional administration of vasopressors, no bleeding, no wound with the possibility of separation, no unstable fracture.				

RASS = Richmond agitation sedation scale; ROM = range of motion; HOB = head of bed; BPS = behavioral pain scale; NRS = numeric rating scale; FIO_2 = fraction of inspiratory oxygen; PEEP = positive end expiratory pressure; EM = early mobilization. [a] This table is cited from reference [1,2]. The EM protocol includes 5 levels: Level 1: head of bed elevation ≤45 degrees and passive range of motion (ROM); Level 2: head of bed elevation ≥60 degrees, active ROM, and continuous lateral rotation therapy; Level 3: sitting on the edge of the bed and rising from the supine position; Level 4: standing at the side of the bed, and standing and pivoting to a chair; and Level 5: walking with assistance and walking independently. Levels 0 and 1 were performed by physical therapists. Level 2 was performed by nurses and physical therapists. Levels 3, 4, and 5 were performed by a team of ICU physicians, nurses and physical therapists.

References

1. Watanabe, S.; Liu, K.; Morita, Y.; Kanaya, T.; Naito, Y.; Arakawa, R.; Suzuki, S.; Katsukawa, H.; Lefor, A.K.; Hasegawa, Y.; et al. Changes in barriers to implementing early mobilization in the intensive care unit: A single center retrospective cohort study. *Nagoya J. Med. Sci.* **2021**, *83*, 443–464.
2. Watanabe, S.; Kotani, T.; Taito, S.; Ota, K.; Ishii, K.; Ono, M.; Katsukawa, H.; Kozu, R.; Morita, Y.; Arakawa, R.; et al. Determinants of gait independence after mechanical ventilation in the intensive care unit: A Japanese multicenter retrospective exploratory cohort study. J. Intensive Care 2019, 7, 53.

Table S3. Details of follow-up.

List	Details
Follow-up period	Between "*two and a half months after discharge*" and "*three and a half months after discharge*" All follow-up assessments were conducted during this one-month period.
Follow-up methods	A person in charge of follow-up at each hospital called the patients and confirmed their survival. Once their survivals were confirmed, the EQ-5D-5L (Japanese version for Japan) was evaluated over the same phone-call.<br><br>Questionnaires and response sheets for HADS and IES-R were then mailed to the patient's home. They answered the questionnaires by themselves or with help of relatives and sent it back to each participating hospital.<br><br>Despite several phone-calls, if it did not reach the patients, the patients were regarded as lost to follow-up and excluded from analysis.
Follow-up person	Follow-up was consistently conducted by the same person at each participating hospital. The person in charge of follow-up could be a doctor, a nurse, or a physiotherapist, depending on the situation of each participating hospital.

EQ-5D-5L = EuroQol-5 Dimensions-5 Levels, HADS = Hospital anxiety and depression scale, IES-R = Impact of event scale-revised.

Table S4. The rationale for collected data.

Long term outcomes	Factors associated with outcomes and collected in this study (references)
Psychiatric symptoms	Age [1], male [1], continuous analgesia [1]<br>mechanical ventilation [2], continuous sedation [2], activities of daily living before hospitalization [3]
Depression	Male [4,5], continuous sedation [4], age [6]. mechanical ventilation [6], admission diagnosis [7], steroids [8], dialysis [9], neuromuscular blocking agents [10]
Anxiety	Mobilization [11]
Post-traumatic stress disorder	Male [4], continuous analgesia [12,13]<br>mechanical ventilation [12,13], continuous sedation [12,13], severity [14]

Long term outcomes	Factors associated with outcomes and collected in this study (references)
Psychiatric symptoms	Age [1], male [1], continuous analgesia [1]<br>mechanical ventilation [2], continuous sedation [2], activities of daily living before hospitalization [3]
Depression	Male [4,5], continuous sedation [4], age [6], mechanical ventilation [6], admission diagnosis [7], steroids [8], dialysis [9], neuromuscular blocking agents [10]
Anxiety	Mobilization [11]
Post-traumatic stress disorder	Male [4], continuous analgesia [12,13]<br>mechanical ventilation [12,13], continuous sedation [12,13], severity [14]

Reference

1. Huang, M.; Parker, A.M.; Bienvenu, O.J.; Dinglas, V.D.; Colantuoni, E.; Hopkins, R.O.; Needham, D.M.; & National Institutes of Health, National Heart, Lung, and Blood Institute Acute Respiratory Distress Syndrome Network. Psychiatric Symptoms in Acute Respiratory Distress Syndrome Survivors: A One-Year National Multi-Center Study. *Crit. Care Med*. **2016**, 44, 954–965.
2. Mikkelsen, M. E.; Christie, J. D.; Lanken, P. N.; Biester, R. C.; Thompson, B. T.; Bellamy, S. L.; Localio, A. R.; Demissie, E.; Hopkins, R. O.; Angus, D. C. The adult respiratory distress syndrome cognitive outcomes study. Long-term neuropsychological function in survivors of acute lung injury. *Am J Respir Crit Care Med*. **2012**, 185, 1307–1315.

3. Shima, N.; Miyamoto, K.; Shibata, M.; Nakashima, T.; Kaneko, M., Shibata, N.; Shima, Y.; Kato, S.; & W-PICS investigators. Activities of daily living status and psychiatric symptoms after discharge from an intensive care unit: a single-center 12-month longitudinal prospective study. *Acute medicine & surgery*. **2020**, *7*(1), e557.
4. LaBuzetta, J. N.; Rosand, J.; Vranceanu, A. M. Review: Post-Intensive Care Syndrome: Unique Challenges in the Neurointensive Care Unit. *Neurocrit Care.* **2019**, 311(3), 534–545.
5. Inoue, S.; Hatakeyama, J.; Kondo, Y.; Hifumi, T.; Sakuramoto, H.; Kawasaki, T.; Taito, S.; Nakamura, K.; Unoki, T.; Kawai, Y.; et al. Post-intensive care syndrome: its pathophysiology, prevention, and future directions. *Acute medicine & surgery.* **2019**, *6*(3), 233–246.
6. Rabiee, A.; Nikayin, S.; Hashem, M. D.; Huang, M.; Dinglas, V. D.; Bienvenu, O. J.; Turnbull, A. E.; Needham, D. M. Depressive Symptoms After Critical Illness: A Systematic Review and Meta-Analysis. *Critical care medicine*. **2016**, *44*(9), 1744–1753.
7. Easton, K.; Coventry, P.; Lovell, K.; Carter, L. A.; Deaton, C. Prevalence and Measurement of Anxiety in Samples of Patients With Heart Failure: Meta-analysis. *The Journal of cardiovascular nursing*. **2016**, *31*(4), 367–379.
8. Kondo, I.; Arai, Y.; Hamada, A.; Yamada, K.; Shioji, S.; Sakamoto, E.; Katagiri, D.; Tada, M.; Hinoshita, F. A Case of Frequently Relapsing Minimal-Change Nephrotic Syndrome with Steroid-Induced Psychiatric Syndrome Treated by Low-Dose, Short-Term Steroid Therapy in Combination with Cyclosporine. *Case reports in nephrology and dialysis*. **2020**, *10*(1), 1–8.
9. Hedayati, S. S.; Finkelstein, F. O. Epidemiology, diagnosis, and management of depression in patients with CKD. *American journal of kidney diseases : the official journal of the National Kidney Foundation*. **2009**, *54*(4), 741–752.
10. Narimatsu, E.; Nakayama, Y.; Sumita, S.; Iwasaki, H.; Fujimura, N.; Satoh, K.; Namiki, A. Sepsis attenuates the intensity of the neuromuscular blocking effect of d-tubocurarine and the antagonistic actions of neostigmine and edrophonium accompanying depression of muscle contractility of the diaphragm. *Acta anaesthesiologica Scandinavica*. **1999**, *43*(2), 196–201.
11. Nikayin, S.; Rabiee, A.; Hashem, M. D.; Huang, M.; Bienvenu, O. J.; Turnbull, A. E.; Needham, D. M. Anxiety symptoms in survivors of critical illness: a systematic review and meta-analysis. *General hospital psychiatry*. **2016**, *43*, 23–29.
12. Caiuby, A. V.; Andreoli, P. B.; Andreoli, S. B. Post-traumatic stress disorder in intensive care unit patients. Transtorno de estresse pós-traumático em pacientes de unidade de terapia intensiva. *Revista Brasileira de terapia intensiva*. **2010**, *22*(1), 77–84.
13. Davydow, D. S.; Gifford, J. M.; Desai, S. V.; Needham, D. M.; Bienvenu, O. J. Posttraumatic stress disorder in general intensive care unit survivors: a systematic review. *General hospital psychiatry*. 2008, *30*(5), 421–434.
14. Tang, F.; Tan, J.; Guo, X.; Huang, J.; Yi, J.; Wang, L. Risk factors for post-traumatic stress disorder in acute trauma patients: A protocol for systematic review and meta-analysis. *Medicine*. 2021, *100*(17), e25616.

Table S5. Details of the post-hoc sensitivity analysis.

A. The inverse probability of treatment weighting statistics, considering the bias related to follow-up loss and death

The inverse probability of treatment weighting (IPTW) method based on propensity scores was applied to outcome factors associated with early mobilization (EM) [1]. This allowed us to adjust for confounding factors between the binary groups, which facilitates an evaluation of causal effects without reducing the sample size by using the estimated propensity scores to construct data weights. Using data from 192 patients discharged from the intensive care unit (ICU), we select the confounders that were potentially associated with the outcome (able to follow up, lost to follow up, death) and calculated propensity scores using logistic regression analysis. Using these weights, the IPTW method based on the propensity score for the 99 patients analyzed in this study was applied to outcome factors associated with early mobilization, in addition to the same covariates used in the multiple analysis of the primary outcome: psychiatric symptoms, including age, male gender, use of mechanical ventilation, use of continuous analgesia, use of continuous sedation. Logistic regression analysis was performed on the basis of the IPTW odds ratios (OR) and confidence intervals (CI).

B. The inverse probability of treatment weighting statistics considering 19 important cofounders potentially affecting the results

We used propensity score to reduce the effects of potential confounding [2] based on 19 variables; age, gender, body mass index, Charlson comorbidity index, Barthel index before hospitalization, ICU admission diagnosis including acute respiratory failure, cardiovascular disease, gastric or colonic surgery, and sepsis non-pulmonary, acute physiology and chronic health evaluation II score, sequential organ failure assessment score at ICU admission, use of mechanical ventilation, use of continuous vasopressor, use of continuous analgesia, use of continuous sedation, use of steroids, use of neuromuscular blocking agents, use of dialysis, average Richmond agitation sedation scale score

during day shift from day 1 to day 3, that were potentially associated with the outcome on the basis of our clinical experience and knowledge. Logistic regression analysis was used to calculate the propensity scores. The IPTW method based on propensity scores was applied to the outcome factors that were associated with EM [3]. This allowed us to adjust for confounding factors between the binary groups, which facilitates an evaluation of causal effects without reducing the sample size by using the estimated propensity scores to construct data weights. Logistic regression analysis was performed on the basis of the IPTW OR and CI.

(Reference)

1. Fukunaga, S.; Nagami, Y.; Shiba, M.; Ominami, M.; Tanigawa, T.; Yamagami, H.; Tanaka, H.; Muguruma, K.; Watanabe, T.; et al. Long-term prognosis of expanded-indication differentiated-type early gastric cancer treated with endoscopic submucosal dissection or surgery using propensity score analysis. *Gastrointestinal endoscopy*. **2017**, *85*(1), 143–152.
2. Nagami, Y.; Tominaga, K.; Machida, H.; Nakatani, M.; Kameda, N.; Sugimori, S.; Okazaki, H.; Tanigawa, T.; Yamagami, H.; Kubo, N.; et al. Usefulness of non-magnifying narrow-band imaging in screening of early esophageal squamous cell carcinoma: a prospective comparative study using propensity score matching. *The American journal of gastroenterology*. **2014**, *109*(6), 845–854.
3. Fukunaga, S.; Nagami, Y.; Shiba, M.; Ominami, M.; Tanigawa, T.; Yamagami, H.; Tanaka, H.; Muguruma, K.; Watanabe, T.; Tominaga, K.; et al. Long-term prognosis of expanded-indication differentiated-type early gastric cancer treated with endoscopic submucosal dissection or surgery using propensity score analysis. *Gastrointestinal endoscopy*. **2017**, *85*(1), 143–152.

Table S6. Comparison of baseline characteristics of patients considering bias related to loss to follow-up and death.

Baseline characteristics	All patients discharged from ICU n= 192	All patients who completed follow up on 3 months n= 99	Early Mobilization group who discharged from ICU n= 107	Early Mobilization group who completed follow up on 3 months n= 60	Non-Early Mobilization group who discharged from ICU n= 85	Non-Early Mobilization group who completed follow up on 3 months n= 39
Age (years), median (IQR)	71 (60–79)	70 (60–78)	70 (61–78)	70 (61–75)	75 (60–81)	73 (57–79)
Gender (male), n (%)	124 (65)	62 (62)	72 (67)	39 (65)	52 (61)	23 (59)
BMI (kg/m^2), median (IQR)	23 (20–26)	23 (21–26)	23 (21–25)	23 (21–25)	23 (20–26)	24 (20–27)
Charlson Comorbidity Index (IQR)	2 (1–3)	2 (0–2)	2 (0–3)	1 (0–2)	2 (0–3)	1 (0–2)
Barthel index before hospitalization, median (IQR) [a]	100 (100–100)	100 (100–100)	100 (100–100)	100 (100–100)	100 (100–100)	100 (100–100)
ICU admission diagnosis, n (%)						
Acute respiratory failure (including pneumonia)	36 (19)	12 (12)	17 (16)	6 (10)	19 (22)	6 (15)
Cardiovascular disease	75 (39)	48 (48)	44 (41)	30 (50)	31 (36)	18 (46)
Gastric or colonic surgery	29 (15)	14 (14)	19 (18)	10 (17)	10 (12)	4 (11)
Sepsis, non-pulmonary	32 (17)	14 (14)	18 (17)	9 (15)	14 (17)	5 (13)
Other diagnoses	20 (10)	11 (11)	9 (8)	5 (8)	11 (13)	6 (15)
APACHE II score, median (IQR)	20 (14–25)	18 (14–26)	18 (13–24)	17 (12–22)	20 (16–26)	21 (16–26)
SOFA at ICU admission, median (IQR)	7 (4–10)	7 (3–10)	6 (3–8)	7 (3–8)	8 (5–11)	7 (4–11)
The use of mechanical ventilation during ICU stays, n (%)	120 (63)	61 (61)	60 (56)	32 (53)	60 (71)	29 (74)
The use of continuous vasopressor during ICU stays, n (%)	109 (57)	59 (59)	53 (50)	34 (57)	56 (66)	25 (64)

The use of continuous analgesia during ICU stays, n (%)	123 (64)	63 (63)	69 (65)	37 (62)	54 (64)	26 (67)
The use of continuous sedation during ICU stay, n (%)	136 (71)	73 (73)	73 (68)	45 (75)	63 (74)	28 (71)
The use of steroids during ICU stays, n (%)	38 (20)	21 (21)	10 (9)	7 (12)	28 (33)	14 (35)
The use of neuromuscular blocking agents during ICU stays, n (%)	10 (5)	5 (5)	1 (1)	0 (0)	9 (11)	5 (13)
The use of dialysis during ICU stay, n (%)	40 (21)	18 (18)	17 (16)	7 (12)	23 (27)	11 (28)
Average RASS score during day shift from day 1 to day 3, median (IQR) [b]	0 (0–0)	0 (0–0)	0 (0–0)	0 (0–0)	0 (0–0)	0 (-2–0)

Data are presented as median (interquartile range) or number (%); IQR = interquartile range; BMI = Body mass index; ICU = Intensive Care Unit; APACHE II = Acute Physiology and Chronic Health Evaluation; SOFA = Sequential Organ Failure Assessment; RASS = Richmond agitation sedation scale. There was no significant difference in all baseline characteristics between patients discharged from the ICU and patients who completed follow up at 3 months, early mobilization group discharged from the ICU and early mobilization group who completed follow up at 3 months, or non-early mobilization group discharged from the ICU and non-early mobilization group who completed follow up at 3 months. [a] Barthel index before hospitalization was scored at the time of ICU admission based on the information from the family or the patients if they were conscious.

[b] In all centers, RASS score, as a sedation scale, was monitored every two hours during the day shift by nurses and recorded in medical record. The best number of RASS, which means the recorded number closest to Zero during the day, of each day from day 1 to 3 was used to calculate the average number of RASS.

Table S7. Association between early mobilization and psychiatric disorder assessment score

Outcomes	**Unadjusted** standardized partial regression coefficient (95%CI)	β coefficient	**Adjusted**[a] standardized partial regression coefficient (95%CI)	β coefficient
At follow up 3 months after hospital discharge				
HADS depression score	0.74 (−0.04–1.53)	0.188	0.44 (−0.34–1.42)	0.137
HADS anxiety score	1.39 (0.73–2.05)	0.391	0.37 (0.36–1.85)	0.311
IES-R score	4.57 (2.49–6.66)	0.404	1.18 (0.79–5.50)	0.278
At hospital discharge				
HADS depression score	0.63 (−0.15–1.41)	0.163	0.61 (−1.21–0.56)	0.083
HADS anxiety score	0.32 (−0.42–1.28)	0.102	0.51 (−1.41–0.63)	0.092
IES-R score	2.53 (0.47–4.59)	0.243	1.21 (−4.11–0.70)	0.164
Changes between follow-up at 3 months and hospital discharge				
HADS depression score	−0.23 (−1.17–0.71)	−0.049	−0.42 (−0.66–1.58)	−0.098
HADS anxiety score	−1.09 (−1.87– -0.31)	−0.271	0.44 (0.08–1.86)	−0.242
IES-R score	2.57 (−0.15–5.28)	−0.217	1.28 (-0.59–4.49)	−0.177

Data are presented as coefficient with 95% confidence interval. *HADS* = Hospital anxiety and depression scale, *IES-R* = Impact of event scale-revised, *CI* = Confidence interval. [a] Multiple linear regression analysis was performed to determine the primary outcome with the covariates. The covariates in the multi-variates analysis included age, male gender, Barthel index before hospitalization, ICU admission diagnosis (acute respiratory failure, cardiovascular disease, gastric or colonic surgery, sepsis, other), acute physiology and chronic health evaluation II score, use of mechanical ventilation, use of continuous analgesia, use of continuous sedation, use of steroids, use of neuromuscular blocking agents, and use of dialysis.

Table S8. Sensitivity analysis using inverse probability of treatment weighted statistics considering bias from death and loss to follow-up.

Outcomes	Adjusted odds ratio (95%CI)	P value
The Primary Outcome		
At the time of follow up on 3 months after hospital discharge		
Patients with psychiatric symptoms[a]	0.28 (0.11–0.73)	0.008
At the time of hospital discharge		
Patients with psychiatric symptoms[a]	0.65 (0.28–1.52)	0.320
Secondary Outcomes		
At follow up at 3 months after hospital discharge		
Patients who scored HADS subset for depression ≥8	0.47 (0.15–1.48)	0.123
HADS subset score for depression	0.21 (0.04–1.11)	0.068
Patients who scored HADS subset for anxiety ≥8	0.22 (0.06–0.85)	0.031
HADS subset score for anxiety	0.08 (0.02–0.31)	0.009
Patients who scored IES-R ≥25	0.53 (0.01–0.66)	0.019
IES-R score	0.01 (0.01–0.03)	0.006
At hospital discharge		
Patients who scored HADS subset for depression ≥8	0.63 (0.27–1.51)	0.296
HADS subset score for depression	0.33 (0.08–1.34)	0.132
Patients who scored HADS subset for anxiety ≥8	1.04 (0.37–2.90)	0.952
HADS subset score for anxiety	0.40 (0.07–2.21)	0.288
Patients who scored IES-R ≥25	0.17 (0.03–0.86)	0.029
IES-R score	0.01 (0.01–0.52)	0.033

Data are presented as coefficient or odds ratio with 95% confidence interval. HADS = Hospital anxiety and depression scale, IES-R = Impact of event scale-revised, EQ-5D-5L = EuroQol-5 Dimensions-5 Levels; CI = Confidence interval. The detail of the analysis is shown in Supplemental Table S2 A. [a] Psychiatric symptoms were defined as the presence of at least one of three symptoms; depression, anxiety, and PTSD.

Table S9. Sensitivity analysis using a propensity score with 19 confounding factors

Outcomes	Odds ratio (95%CI)	P value
Primary Outcome		
At follow up at 3 months after hospital discharge		
Patients who demonstrated psychiatric symptoms[a]	0.49 (0.14–0.93)	0.046
At hospital discharge		
Patients who demonstrated psychiatric symptoms[a]	0.93 (0.32–2.67)	0.893
Secondary Outcomes		
At follow up at 3 months after hospital discharge		
Patients who scored HADS subset for depression ≥8	0.41 (0.14–1.23)	0.108
HADS subset score for depression	0.28 (0.03–2.41)	0.252
Patients who scored HADS subset for anxiety ≥8	0.35 (0.10–1.25)	0.113
HADS subset score for anxiety	0.10 (0.03–0.39)	< 0.001
Patients who scored IES-R ≥25	0.07 (0.01–0.63)	0.011
IES-R score	0.01 (0.01–0.46)	0.016
At hospital discharge		
Patients who scored HADS subset for depression ≥8	0.78 (0.26–2.35)	0.647
HADS subset score for depression	0.61 (0.09–4.32)	0.622
Patients who scored HADS subset for anxiety ≥8	1.42 (0.44–4.57)	0.545
HADS subset score for anxiety	0.77 (0.13–4.58)	0.783
Patients who scored IES-R ≥25	0.23 (0.04–1.36)	0.101
IES-R score	0.03 (0.01–3.30)	0.137

Data are presented as coefficient or odds ratio with 95% confidence interval. HADS = Hospital anxiety and depression scale, IES-R = Impact of event scale-revised, EQ-5D-5L = EuroQol-5 Dimensions-5 Levels; RR=Risk ratio; CI=Confidence interval. The detail of the analysis is shown in Supplemental Table S2 B.

[a] Psychiatric symptoms were defined as the presence of at least one of three symptoms; depression, anxiety, and PTSD.
